# Enterprise AI applications and stock price crash risk

**DOI:** 10.3389/frai.2026.1914515

**Published:** 2026-07-22

**Authors:** Zhao Yang

**Affiliations:** Jining University, Jining, China

**Keywords:** artificial intelligence, CEO’S digital expertise, dual-channel mechanism, financial market stability, stock price crash risk

## Abstract

With Chinese A-share listed enterprises (2015–2024), this paper studies the influence of artificial intelligence applications on stock price crash risk. Our conclusion includes that AI applications significantly reduce the crash risk. The mechanism analysis reveals that artificial intelligence works by improving internal controls quality (ICQ) and decision-making efficiency (DME). Heterogeneity tests show the effect is stronger for enterprises with digital expertise among CEO and located in eastern China. This paper provides a theoretical basis for enterprises to optimize their development strategies and prevent capital market risk.

## 1 Introduction

The stock price crash risk means the risk of inducing a sharp plunge in stock prices due to information asymmetry and concealing adverse news to the limit, which will reverse investor expectations and damage market confidence, and even influence the security of the financial system. Recently, the ability of AI to break through traditional management paradigms in data processing, knowledge creation, and intelligent decision-making is reshaping enterprise risk. Thus, exploring the financial value and influence mechanism of AI has become an important topic to regulate the development of capital markets.

The extant literature on AI focuses on two primary dimensions: qualitative theoretical interpretations and quantitative analyses of economic consequences. From a theoretical perspective, AI, as a new production factor in digital form, has both tool carrier and applications object attributes, and can be integrated into market allocation through R&D transformation (Yang, 2026). Compared with traditional elements, AI is non-competitive and can simultaneously exert performance in multiple scenarios and multiple subjects (Zhou and He, 2026). In terms of economic consequences, the influence of AI has been explored at both the city and business levels. At the city level, AI has a significant influence on entrepreneurial activity (Fang et al., 2025; Duong et al., 2026), urban resilience (Deng et al., 2023; Neng et al., 2026) and regional innovation (Zheng et al., 2025; Deng, 2025); At the enterprise level, AI affects resource allocation efficiency (Sun and Zhou, 2025), enterprise innovation (Li et al., 2023; Zhong and Song, 2025; Feng et al., 2025; Zhang and Li, 2026) and new quality productivity (Chin et al., 2025; Chen et al., 2025).

Collectively, this literature centers around the real economic market, and there is still a lack of a clear understanding of the influence of AI on the financial domain. Therefore, based on Chinese A-share listed enterprises (2015–2024), this paper centers on exploring the influence of AI on the stock price crash risk. The possible contributions: (1) incorporating AI into the stock price crash risk analysis framework, explaining its prevention and control path, and expanding the existing research horizon; (2) clarify the empowerment mechanism of AI from the dual dimensions of supervision intensity and decision-making efficiency, and provide a new theoretical perspective for intelligent governance; (3) based on the heterogeneity of the influence of CEO digital expertise and geographical location, it provides micro-empirical support for accurately preventing crash risk.

## 2 Hypotheses

### 2.1 AI and stock price crash risk

Based on the “management cover-up hypothesis,” negative information hoarding is the core cause of stock price crashes, while AI can optimize the enterprise information environment by reshaping the information disclosure model. In terms of internal governance, intelligent technology ensures the authenticity of accounting information through the coupling of business and information flow. In terms of external supervision, AI achieves full penetration of supervision by reducing information processing costs. This internal and external linkage mechanism effectively disintegrates the management’s motivation to “cover the market,” thereby systematically curbing the crash risk. In light of this, a hypothesis will be:

*H1*: AI can reduce the stock price crash risk.

### 2.2 Mechanism

#### 2.2.1 The ICQ mechanism

Enterprise AI applications reconfigure internal control frameworks through governance mechanisms distinct from traditional digital transformation. While traditional digital technologies function as passive, rule-based recording tools that enhance linear transparency, AI introduces cognitive, predictive capabilities. By deploying machine learning, AI integrates fragmented, unstructured data streams to autonomously extract complex, non-linear risk features that typically evade standard filters.

This technological distinctiveness shifts internal control from post-event manual compliance auditing to autonomous, real-time risk pre-warning (Atta, 2025), effectively detecting concealed related-party transactions and anomalous cash flows at their inception (Chen et al., 2023). Consequently, this AI-driven mechanism represents a structural “governance-hardening” effect rather than mere information disclosure. By severely curtailing management’s capacity to strategically hoard negative news, AI suppresses the source of hidden corporate misconduct (Yang and Zhou, 2025; Zhang et al., 2025), thereby preventing sudden capital market panics and mitigating stock price crash risk. In light of this, a hypothesis will be:

*H2a*: AI enhances ICQ to mitigate stock price crash risk.

#### 2.2.2 The DME mechanism

Enterprise AI applications process operational data, macroeconomic variables, and industry trends in real time, providing predictive decision support. Compared to traditional digital systems, AI uses deep learning algorithms to identify nonlinear causal relationships in historical data, simulate multiple decision paths under complex scenarios, and optimize resource allocation plans (Agrawal et al., 2019). Through forward-looking decision support, enterprises can detect signals of business volatility, improve the efficiency of strategic decision-making, and avoid performance fluctuations caused by blind decision-making (Pu et al., 2025; Hemphill, 2019). When enterprises use AI-generated, precise recommendations to adjust their business direction and align with market demand, the stability of profitability and the transparency of financial disclosures are ensured, which helps reduce information asymmetry between internal managers and external stakeholders (Li et al., 2025). By strengthening operational fundamentals, this approach can cushion potential performance shocks and fundamentally mitigate stock price crash risk caused by irrational expectations (Chen et al., 2026). In light of this, a hypothesis will be:

*H2b*: AI enhances decision-making efficiency, thereby mitigating stock price crash risk.

## 3 Research design

### 3.1 Sample description

This paper selects Chinese A-share listed enterprises 2015–2024 as the research sample, and subjects the sample to the following treatments: (1) Exclude samples with missing values for key variables;(2) Exclude the financial industry sample; (3) Exclude samples designated as ST, *ST, or PT. Winsorize continuous variables at the 1 and 99% quantiles. The primary data source is the Wind database (WIND) and the Guotai An database (CSMAR).

### 3.2 Variable description

#### 3.2.1 Explained variable

Stock price crash risk is the explained variable. Drawing from the literature (Sun et al., 2025), we take the negative conditional return skewness (Ncskew) and the down-to-up volatility ratio (Duvol) as the explained variable. The higher the values of both, the greater the intensity of crash risk.

#### 3.2.2 Explanatory variable

We measured the AI adoption based on the text of enterprise annual reports. When constructing the base vocabulary, we referred to AI industry reports from iResearch and Deloitte, and, by combining these with the characteristics of the annual report text, identified standardized terminology across five dimensions, including technology R&D and industry applications. During the semantic expansion phase, the annual report texts underwent precise word segmentation to remove financial stop words. The Skip-gram architecture of the Word2Vec model was used for term expansion, with a cosine similarity threshold of 0.75 set for filtering. To ensure the professional validity of the terminology, a “machine pre-screening + human audit” refinement process was implemented. Specifically, a 30% stratified sample was drawn from the expanded term set, and two researchers independently evaluated the terms against industry classification standards, with a third party resolving any discrepancies. Regarding metric calculation, the total word frequency of all AI terms in each annual report was summed, and the metrics were log-transformed using ln (frequency+1) to eliminate potential heteroscedasticity.

#### 3.2.3 Control variables

Given that crash risk is subject to more other influences, this paper controls for a range of factors, such as enterprise-level operating traits and governance mechanisms, within the empirical model. The connotation of control variables is detailed in Table 1.

**Table 1:** Variable connotation.

Variable	Definition
Ret	The average weekly rate of return
Sigma	The standard deviation of the annual weekly rate of return
Age	LN (current year–year of establishment +1)
Size	Natural logarithm of total assets
Roe	Net profit/shareholders’ equity
Lev	Total liabilities/total assets
BM	Book-to-market ratio
Cash	Operating cash flow/operating income
Top1	The number of shares held by the largest shareholder/total number of shares
Inst	The shareholding ratio of institutional investors
Big4	Indicator equal to one if the enterprise is audited by a Big Four accounting enterprise in year t, and zero otherwise

### 3.3 Model design

We estimate the following baseline model:


$$\text{NCSKE}W_{i,t+1}=\delta_{0}+\delta_{1}AI_{i,t}+\sum \gamma_{k}\text{Contro}l_{i,t}+\mu_{i}+\gamma_{t}+\varepsilon_{i,t}$$ (1)

$$\text{DUVO}L_{i,t+1}=\delta_{0}+\delta_{1}AI_{i,t}+\sum \gamma_{k}\text{Contro}l_{i,t}+\mu_{i}+\gamma_{t}+\varepsilon_{i,t}$$ (2)

In models (1) and (2), $\text{NCSKE}W_{i,t+1}$ and $\text{DUVO}L_{i,t+1}$ are the explained variable. ${\delta_{1}}^{’}$ s symbol and significance level reflect the influence of AI on the explained variable. $\text{Contro}l_{i,t}$ represents control variables, and $\gamma_{k}$ is the control variable coefficient. In addition, we include individual fixed effects ( $\mu_{i}$ ) and year fixed effects ( $\gamma_{t}$ ). Table 2 presents descriptive statistics for variables.

**Table 2:** Descriptive statistics.

Variable	*N*	Mean	SD	Min	Max
NCSKEW	30,861	−0.256	0.764	−5.25	6.235
DUVOL	30,861	−0.164	0.498	−2.777	3.748
AI	30,861	0.653	1.089	0	6.277
Ret	30,861	0.005	0.189	−0.013	0.137
Sigma	30,861	0.076	1.326	0	1.264
Age	30,861	1.976	0.912	0	3.497
Size	30,861	16.913	1.53	10.842	31.31
Roe	30,861	0.036	1.184	−4.895	3.831
Lev	30,861	0.507	3.932	0.001	0.999
BM	30,861	0.624	0.253	0.064	1.246
Cash	30,861	0.202	0.158	0	1
Top1	30,861	35.632	15.943	0.29	93.1
Inst	30,861	43.789	24.91	0.101	95.636
Big4	30,861	0.06	0.238	0	1

## 4 Empirical results

### 4.1 Baseline regression

Table 3 reports the baseline regression results. Clearly, the coefficients of AI are significantly negative at the 1% level, that is, AI will significantly reduce stock price crash risk, which means the hypothesis H1is verified. Taking columns (3) and (4) as examples, for each additional unit of the AI index, the NCSKEW coefficient decreases by 0.024, and the DUVOL coefficient decreases by 0.018.

**Table 3:** Basic regression.

Variable	(1)	(2)	(3)	(4)
	NCSKEW	DUVOL	NCSKEW	DUVOL
AI	−0.020^***^	−0.015^***^	−0.020^**^	−0.015^***^
	(0.007)	(0.004)	(0.008)	(0.005)
Ret			−9.955^***^	−8.893^***^
			(0.805)	(0.512)
Sigma			−5.685^***^	−2.621^***^
			(0.267)	(0.170)
Age			−0.219^***^	−0.147^***^
			(0.025)	(0.016)
Size			0.036^**^	−0.009
			(0.015)	(0.010)
Roe			−0.075^**^	−0.041^*^
			(0.033)	(0.021)
Lev			−0.038	0.012
			(0.055)	(0.035)
BM			−0.166^***^	−0.001
			(0.044)	(0.028)
Cash			0.169^***^	0.133^***^
			(0.057)	(0.036)
Top1			−0.001	−0.000
			(0.001)	(0.001)
Inst			0.003^***^	0.002^***^
			(0.001)	(0.000)
Big4			−0.014	−0.019
			(0.046)	(0.029)
_cons	−0.300^***^	−0.188^***^	−0.274	0.411^**^
	(0.007)	(0.004)	(0.311)	(0.198)
Stkcd	Y	Y	Y	Y
Year	Y	Y	Y	Y
*N*	30,861	30,861	30,861	30,861
Adj. *R*^2^	0.063	0.070	0.107	0.115

The brackets are robust standard errors, and ^*^, ^**^, and ^***^ represent the significance levels of 10, 5, and 1%, respectively. The same below.

### 4.2 Robustness test

#### 4.2.1 Recalculate the explained variable

In Table 3, the explained variable is calculated based on market returns derived using the weighted-average method based on aggregate market tradable capitalization; for the robustness checks, we employ the weighted-average method based on aggregate market total capitalization for calculation and verification, and in Table 4, the regression estimates are displayed in columns (1) and (2).

**Table 4:** Robustness test_PartA.

Variable	Recalculate the explained variable		Recalculate the explanatory variable					
			AI_inv		AI_rec		AI_patent	
	(1)	(2)	(3)	(4)	(5)	(6)	(7)	(8)
	NCSKEW	DUVOL	NCSKEW	DUVOL	NCSKEW	DUVOL	NCSKEW	DUVOL
AI	−0.021^**^	−0.015^***^						
	(0.008)	(0.005)						
AI_inv			−0.021^**^	−0.016^***^				
			(0.010)	(0.006)				
AI_rec					−0.021^**^	−0.016^**^		
					(0.010)	(0.006)		
AI_patent							−0.022^**^	−0.020^***^
							(0.011)	(0.007)
Ret	−10.635^***^	−9.322^***^	−10.310^***^	−9.004^***^	−14.489^***^	−11.866^***^	−15.506^***^	−12.591^***^
	(0.797)	(0.510)	(0.883)	(0.560)	(0.963)	(0.604)	(1.027)	(0.642)
Sigma	−5.428^***^	−2.519^***^	−5.694^***^	−2.664^***^	−5.993^***^	−2.681^***^	−7.185^***^	−3.310^***^
	(0.265)	(0.169)	(0.297)	(0.188)	(0.326)	(0.204)	(0.359)	(0.225)
Age	−0.238^***^	−0.164^***^	−0.198^***^	−0.127^***^	−0.210^***^	−0.132^***^	−0.232^***^	−0.137^***^
	(0.025)	(0.016)	(0.030)	(0.019)	(0.033)	(0.021)	(0.038)	(0.023)
Size	0.036^**^	−0.006	0.055^***^	0.004	0.053^**^	−0.002	0.061^**^	−0.008
	(0.015)	(0.010)	(0.019)	(0.012)	(0.022)	(0.014)	(0.026)	(0.016)
Roe	−0.074^**^	−0.047^**^	−0.068^*^	−0.039^*^	−0.024	−0.002	−0.012	0.010
	(0.033)	(0.021)	(0.036)	(0.023)	(0.039)	(0.025)	(0.042)	(0.026)
Lev	−0.049	−0.008	−0.039	0.015	0.016	0.074	0.085	0.112^**^
	(0.055)	(0.035)	(0.067)	(0.042)	(0.075)	(0.047)	(0.085)	(0.053)
BM	−0.167^***^	−0.009	−0.111^**^	0.016	−0.105^*^	0.029	−0.148^**^	0.025
	(0.043)	(0.028)	(0.050)	(0.032)	(0.055)	(0.035)	(0.062)	(0.039)
Cash	0.169^***^	0.126^***^	0.153^**^	0.111^***^	0.142^**^	0.117^***^	0.129^*^	0.086^*^
	(0.056)	(0.036)	(0.066)	(0.042)	(0.071)	(0.045)	(0.078)	(0.049)
Top1	−0.001	−0.000	0.001	0.001	0.002	0.001	0.002	0.002^*^
	(0.001)	(0.001)	(0.001)	(0.001)	(0.001)	(0.001)	(0.002)	(0.001)
Inst	0.003^***^	0.002^***^	0.002^***^	0.001^**^	0.003^***^	0.002^***^	0.002^**^	0.001^**^
	(0.001)	(0.000)	(0.001)	(0.000)	(0.001)	(0.001)	(0.001)	(0.001)
Big4	−0.012	−0.018	−0.046	−0.048	−0.050	−0.039	−0.045	−0.050
	(0.046)	(0.029)	(0.054)	(0.034)	(0.059)	(0.037)	(0.067)	(0.042)
_cons	−0.220	0.425^**^	−0.768^*^	0.069	−0.792^*^	0.133	−0.805	0.337
	(0.308)	(0.197)	(0.400)	(0.253)	(0.461)	(0.290)	(0.528)	(0.330)
Stkcd	Y	Y	Y	Y	Y	Y	Y	Y
Year	Y	Y	Y	Y	Y	Y	Y	Y
*N*	30,861	30,861	30,861	30,861	30,861	30,861	30,861	30,861
Adj. *R*^2^	0.110	0.119	0.105	0.111	0.120	0.127	0.127	0.133

#### 4.2.2 Recalculate the explanatory variable

We employ three distinct proxy indicators to replace the existing enterprise AI application indicators derived from annual report texts for regression analysis: firstly, the ratio of AI investment to total enterprise assets (AI_inv), calculated by aggregating the total specialized expenditure on intelligent equipment, algorithm R&D and computing power infrastructure from annual reports and dividing this by total assets at the end of the period; secondly, the scale of AI job recruitment (AI_rec), determined by identifying AI-related positions—such as those involving text recognition algorithms, large language models and smart manufacturing—in enterprises’ annual recruitment announcements and tallying the annual number of recruits; thirdly, the total volume of AI patent applications (AI_patent), calculated by extracting the number of applications (in thousands) for AI sub-technologies—such as machine learning, visual inspection and intelligent production—from the enterprise’s invention and utility model patents filed during the year. Columns (3)–(8) of Table 4 present the estimation results.

#### 4.2.3 Exclude special samples

Compared with other listed enterprises, ChiNext-listed enterprises are characterized by higher technological intensity and a stronger willingness and lower barriers to adopting AI technologies. To prevent these enterprises from biasing the empirical results, we exclude them from the full sample for further testing. The regression estimates are displayed in Table 5 columns (1)–(2).

**Table 5:** Robustness test_PartB.

Variable	Exclude special samples						Fixed Effects for Interaction Terms		Adding control variables	
	Exclude samples of companies listed on the ChiNext board		Exclude samples from the year 2015 of stock market crashes		Exclude samples from the years 2020–2022 affected by the COVID-19 pandemic.					
	(1)	(2)	(3)	(4)	(5)	(6)	(7)	(8)	(9)	(10)
	NCSKEW	DUVOL	NCSKEW	DUVOL	NCSKEW	DUVOL	NCSKEW	DUVOL	NCSKEW	DUVOL
AI	−0.021^**^	−0.018^***^	−0.029^***^	−0.021^***^	−0.021^**^	−0.016^***^	−0.019^**^	−0.015^**^	−0.024^***^	−0.017^***^
	(0.009)	(0.006)	(0.009)	(0.006)	(0.009)	(0.005)	(0.009)	(0.006)	(0.008)	(0.005)
Ret	−10.445^***^	−8.988^***^	−12.317^***^	−9.990^***^	−10.978^***^	−9.620^***^	−10.003^***^	−8.798^***^	−11.713^***^	−10.006^***^
	(0.907)	(0.577)	(0.871)	(0.556)	(0.836)	(0.533)	(0.869)	(0.553)	(0.828)	(0.530)
Sigma	−5.765^***^	−2.629^***^	−4.929^***^	−2.284^***^	−5.515^***^	−2.488^***^	−5.766^***^	−2.701^***^	−4.871^***^	−2.126^***^
	(0.300)	(0.191)	(0.287)	(0.183)	(0.276)	(0.176)	(0.289)	(0.184)	(0.271)	(0.174)
Age	−0.232^***^	−0.157^***^	−0.190^***^	−0.117^***^	−0.204^***^	−0.133^***^	−0.206^***^	−0.140^***^	−0.172^***^	−0.104^***^
	(0.028)	(0.018)	(0.026)	(0.017)	(0.025)	(0.016)	(0.028)	(0.018)	(0.025)	(0.016)
Size	0.037^**^	−0.008	0.043^***^	−0.001	0.032^**^	−0.010	0.039^**^	−0.010	0.015	−0.014
	(0.017)	(0.011)	(0.016)	(0.010)	(0.015)	(0.010)	(0.017)	(0.011)	(0.017)	(0.011)
Roe	−0.097^**^	−0.063^**^	−0.061^*^	−0.035	−0.073^**^	−0.034	−0.068^*^	−0.037	−0.032	−0.015
	(0.040)	(0.025)	(0.036)	(0.023)	(0.036)	(0.023)	(0.036)	(0.023)	(0.036)	(0.023)
Lev	−0.010	0.019	−0.056	−0.022	−0.049	−0.001	−0.038	0.012	−0.048	−0.016
	(0.064)	(0.041)	(0.058)	(0.037)	(0.056)	(0.036)	(0.061)	(0.039)	(0.057)	(0.037)
BM	−0.158^***^	0.002	−0.187^***^	−0.025	−0.176^***^	−0.015	−0.212^***^	−0.026	−0.131^***^	0.001
	(0.049)	(0.031)	(0.046)	(0.029)	(0.044)	(0.028)	(0.047)	(0.030)	(0.044)	(0.028)
Cash	0.133^**^	0.115^***^	0.142^**^	0.129^***^	0.187^***^	0.151^***^	0.156^**^	0.124^***^	0.181^***^	0.136^***^
	(0.062)	(0.040)	(0.060)	(0.038)	(0.057)	(0.036)	(0.062)	(0.039)	(0.058)	(0.037)
Top1	−0.001	0.000	−0.000	−0.000	−0.002	−0.001	−0.000	0.000	−0.002	−0.001
	(0.001)	(0.001)	(0.001)	(0.001)	(0.001)	(0.001)	(0.001)	(0.001)	(0.001)	(0.001)
Inst	0.004^***^	0.002^***^	0.004^***^	0.002^***^	0.004^***^	0.002^***^	0.004^***^	0.002^***^	0.004^***^	0.003^***^
	(0.001)	(0.000)	(0.001)	(0.000)	(0.001)	(0.000)	(0.001)	(0.000)	(0.001)	(0.000)
Big4	−0.058	−0.043	0.003	0.002	−0.047	−0.033	−0.011	−0.009	−0.021	−0.022
	(0.052)	(0.033)	(0.052)	(0.033)	(0.046)	(0.029)	(0.049)	(0.031)	(0.049)	(0.031)
Digital									−0.105^*^	−0.085^***^
									(0.062)	(0.026)
Analyst									−0.192^***^	−0.088^***^
									(0.054)	(0.022)
Media									−0.085	−0.085^***^
									(0.063)	(0.025)
Consv									−0.892^***^	0.098
									(0.322)	(0.120)
Opacity									0.867	0.396
									(1.028)	(0.303)
_cons	−0.284	0.384^*^	−0.528^*^	0.167	−0.220	0.401^**^	−0.357	0.433^**^	−0.057	0.367
	(0.362)	(0.230)	(0.318)	(0.203)	(0.312)	(0.199)	(0.342)	(0.218)	(0.350)	(0.224)
Stkcd	Y	Y	Y	Y	Y	Y	Y	Y	Y	Y
Year	Y	Y	Y	Y	Y	Y	Y	Y	Y	Y
Industry * Year							Y	Y		
City * Year							Y	Y		
*N*	23,935	23,935	27,775	27,775	19,278	19,278	30,861	30,861	30,861	30,861
r2_a	0.111	0.117	0.103	0.109	0.110	0.116	0.106	0.111	0.108	0.113

#### 4.2.4 Add fixed effects for interaction terms

To eliminate estimation biases arising from the omission of time-varying shocks, such as annual sector-specific policies and regional industrial support measures, we add industry -year and city–year interaction fixed effects, and the regression was re-run. The regression estimates are displayed in Table 5 columns (3)–(4).

#### 4.2.5 Adding control variables

To mitigate the bias arising from omitted variables and accurately isolate the independent effect of artificial intelligence applications, this paper sequentially introduces five control variables—digital transformation (Digital), analyst coverage (Analyst), media attention (Media), accounting conservatism (Consv), and enterprise opacity (Opacity)—for testing. The digital transformation indicator (Digital) is constructed based on the number of digital invention patent applications filed by enterprises. Given that the implementation of artificial intelligence is merely a component of a company’s overall digital transformation, the model is highly prone to overestimating the economic impact of AI technology itself if the overall level of digital development is not controlled for; analyst coverage (Analyst) is defined as the total number of analysts tracking the listed company in a given year; media attention (Media) is calculated as the number of full-text news articles related to the company published by financial media outlets during the year; Accounting conservatism (Consv) is measured using the robustness coefficient derived from the classic Basu model; Enterprise information opacity (Opacity) is measured using the average of the absolute values of manipulable accruals over three consecutive years. The aforementioned variables are capable of isolating the interference of confounding factors—such as the broader digital development environment, external supervision by capital markets, media scrutiny, enterprise accounting practices, and distortions in information disclosure—on the dependent variable, thereby reducing estimation bias in the model and enabling the regression results to reflect the true marginal impact of AI applications more objectively. The regression estimates are displayed in Table 5 columns (5)–(6).

The above regression estimates support that AI can significantly reduce crash risk, considering robustness.

### 4.3 Endogenous test

#### 4.3.1 Instrumental variable method

Given that enterprise AI transformation may be subject to unobserved individual heterogeneity or bidirectional causality, this paper follows the approach of Song et al. (2026) and employs the Bartik method—which combines a historical base period with a national trend—to construct an instrumental variable (IV). Specifically, the IV is measured using the product of the number of telephone lines per 10,000 people in the province where the firm is registered in 2002 and the number of Internet users nationwide in that year. This variable satisfies both core conditions for an instrumental variable: on the one hand, regions with more developed historical traditional telecommunications infrastructure possess more mature digital ecosystems and information technology capabilities, which can significantly reduce the marginal cost of deploying AI technology for individual enterprise today, thereby fulfilling the correlation assumption of high correlation with the endogenous variable; on the other hand, provincial telephone penetration rates from decades ago constitute historical facts beyond the control of enterprises; they are not only highly exogenous in terms of time but also have absolutely no possibility of directly influencing the risk of stock price collapses for micro-enterprises in the current capital market across time and space. They can only exert an effect through the specific channel of “promoting the current adoption of AI by local enterprises,” thereby strictly satisfying the exogeneity assumption regarding the exclusionary constraint. The regression estimates, in Table 5 Column (1)–(3), indicate that the key conclusions remain robust even accounting for potential reverse causality.

#### 4.3.2 Policy shock

In 2019, China’s Ministry of Science and Technology launched the ‘National New Generation AI Innovation and Development Pilot Zones’ initiative, establishing a total of 18 pilot zones across the country in 2019 and 2020. Through the policy guidance and concentration of resources provided by these pilot zones, enterprises have been able to accelerate their exploration of artificial intelligence application scenarios. To further examine the dynamic economic effects driven by these policies, this paper constructs a policy shock test to investigate the impact of enterprises’ artificial intelligence applications on stock price crashes. The testing model is shown in models (3) and (4), where is the core explanatory variable $\text{Trea}t_{i}\times \mathrm{Pos}t_{t}$ in this section, indicating whether the city in which the enterprise is located was included in the National Pilot Zones in a given year. If included, this interaction term takes the value 1; otherwise, it takes the value 0.


$$\begin{array}{c} \text{NCSKE}W_{i,t+1}=\delta_{0}+\delta_{1}\text{Trea}t_{i}\times \mathrm{Pos}t_{t}\\ +\sum \gamma_{k}\text{Contro}l_{i,t}+\mu_{i}+\gamma_{t}+\varepsilon_{i,t} \end{array}$$ (3)

$$\begin{array}{c} \text{DUVO}L_{i,t+1}=\delta_{0}+\delta_{1}\text{Trea}t_{i}\times \mathrm{Pos}t_{t}\\ +\sum \gamma_{k}\text{Contro}l_{i,t}+\mu_{i}+\gamma_{t}+\varepsilon_{i,t} \end{array}$$ (4)

Columns (5) and (6) of Table 6 present the regression results. It is evident that the coefficient for “ $\text{Trea}t_{i}\times \mathrm{Pos}{t_{t}}^{”}$ is significantly negative at the 1% significance level, indicating that AI has a negative, mitigating effect on the crash risk. The estimation results are consistent with the conclusions of the baseline regression.

**Table 6:** Endogenous test.

Variable	Instrumental variable method			Policy shock			
	Stage 1	Stage 2					
	(1)	(2)	(3)	(4)	(5)	(6)	(7)
	AI	NCSKEW	DUVOL	NCSKEW	DUVOL	NCSKEW	DUVOL
IV	0.860^***^						
	(0.005)						
AI		−0.025^***^	−0.010^***^				
		(0.006)	(0.004)				
Treat × Post				−0.073^***^	−0.051^***^		
				(0.016)	(0.009)		
pre5						−0.025	−0.002
						(0.019)	(0.011)
pre4						0.005	0.011
						(0.014)	(0.008)
pre3						−0.008	0.001
						(0.012)	(0.006)
pre2						0.003	0.005
						(0.008)	(0.004)
current						−0.030^***^	−0.019^***^
						(0.009)	(0.005)
post1						−0.089^***^	−0.055^***^
						(0.024)	(0.014)
post2						−0.101^***^	−0.063^***^
						(0.016)	(0.009)
post3						−0.129^***^	−0.082^***^
						(0.022)	(0.012)
post4						−0.126^***^	−0.086^***^
						(0.031)	(0.016)
post5						−0.221^***^	−0.144^***^
						(0.044)	(0.024)
Ret	−7.129^***^	−9.233^***^	−6.783^***^	2.621^***^	1.415^***^	3.086^***^	2.291^***^
	(0.611)	(0.664)	(0.421)	(0.599)	(0.378)	(0.592)	(0.374)
Sigma	1.695^***^	−3.780^***^	−2.077^***^	−0.842^***^	−0.413^***^	−0.827^***^	−0.763^***^
	(0.205)	(0.222)	(0.141)	(0.229)	(0.145)	(0.226)	(0.143)
Age	−0.100^***^	−0.106^***^	−0.066^***^	−0.110^***^	−0.072^***^	−0.272^***^	−0.187^***^
	(0.007)	(0.008)	(0.005)	(0.026)	(0.017)	(0.026)	(0.016)
Size	0.046^***^	0.005	−0.017^***^	0.005	−0.009	0.173^***^	0.094^***^
	(0.005)	(0.006)	(0.004)	(0.016)	(0.010)	(0.016)	(0.010)
Roe	0.064^**^	−0.008	−0.013	0.039	0.018	−0.163^***^	−0.117^***^
	(0.027)	(0.029)	(0.019)	(0.035)	(0.022)	(0.034)	(0.022)
Lev	−0.003	−0.004	0.002	−0.067	−0.023	−0.289^***^	−0.168^***^
	(0.027)	(0.029)	(0.019)	(0.059)	(0.037)	(0.058)	(0.036)
BM	−0.160^***^	−0.245^***^	−0.073^***^	−0.110^**^	−0.065^**^	−0.653^***^	−0.381^***^
	(0.023)	(0.025)	(0.016)	(0.044)	(0.027)	(0.043)	(0.027)
Cash	0.395^***^	−0.013	0.012	0.038	0.021	0.026	0.023
	(0.036)	(0.039)	(0.025)	(0.060)	(0.038)	(0.059)	(0.037)
Top1	−0.002^***^	−0.002^***^	−0.001^***^	0.002^*^	0.000	0.000	−0.001
	(0.000)	(0.000)	(0.000)	(0.001)	(0.001)	(0.001)	(0.001)
Inst	−0.001^***^	0.000	0.000	0.002^**^	0.001^***^	0.002^***^	0.001^***^
	(0.000)	(0.000)	(0.000)	(0.001)	(0.000)	(0.001)	(0.000)
Big4	0.009	−0.053^***^	−0.038^***^	−0.004	0.011	−0.004	−0.016
	(0.019)	(0.021)	(0.013)	(0.048)	(0.031)	(0.048)	(0.030)
_cons	−0.475^***^	0.291^***^	0.537^***^	−0.156	0.173	−3.110^***^	−1.597^***^
	(0.103)	(0.111)	(0.071)	(0.330)	(0.208)	(0.325)	(0.205)
Stkcd	Y	Y	Y	Y	Y	Y	Y
Year	Y	Y	Y	Y	Y	Y	Y
*N*	30,861	30,861	30,861	30,861	30,861	30,861	30,861
Adj. *R*^2^	0.613	0.127	0.132	0.069	0.075	0.074	0.073
Cragg–Donald Wald F	130.814 [16.38]						
Klei Bergen–Paaprk LM	6.261^**^						

**Figure 1:**
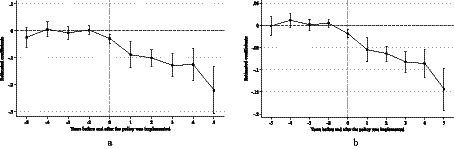
**(a)** Parallel Trend_NCSKEW, **(b)** Parallel Trends_DUVOL.

To test whether the treatment and control groups satisfied the parallel trends assumption before policy implementation, this study also constructed a dynamic effects evaluation model [as shown in models (5) and (6)] for testing, with the results presented in Figures 1a,b, Columns (7) and (8) of Table 6. Taking the year of policy implementation (*t* = 0) as the base period, for the period ranging from 5 to 2 years before policy implementation (*t* = −5 to *t* = −2), the estimated regression coefficients for each period all fall within the 95% confidence interval around zero. This indicates that, prior to the implementation of the ‘National New Generation AI Innovation and Development Pilot Zones’ policy, there was no significant difference between the treatment group and the control group in the trend of share price crash risk, thereby passing the parallel trends test. In terms of the dynamic effects following policy implementation, in the year of policy implementation (*t* = 0) and the subsequent years (*t* = 1 to *t* = 4), the estimated regression coefficients were all significantly negative and exhibited a sustained downward trend, indicating that the mitigating effect of AI on the crash risk is markedly persistent. The aforementioned analysis of dynamic effects not only validates the parallel trends hypothesis but also further corroborates the reliability of the baseline regression results.


$$\begin{array}{c} \text{NCSKE}W_{i,t+1}=\delta_{0}+\sum_{k=-5,k\neq -1}^{4}\beta_{k}\text{Trea}t_{i}\times \mathrm{Yea}r_{t,k}\\ +\sum \gamma_{k}\text{Contro}l_{i,t}+\mu_{i}+\gamma_{t}+\varepsilon_{i,t} \end{array}$$ (5)

$$\begin{array}{c} \text{DUVO}L_{i,t+1}=\delta_{0}+\sum_{k=-5,k\neq -1}^{4}\beta_{k}\text{Trea}t_{i}\times \mathrm{Yea}r_{t,k}+\sum \gamma_{k}\text{Contro}l_{i,t}\\ +\mu_{i}+\gamma_{t}+\varepsilon_{i,t} \end{array}$$ (6)

### 4.4 Mechanism of action test

Building on models (1) and (2), we construct the following empirical models to further explore the specific mechanisms through which improvements in ICQ and DME influence the relationship between AI and stock price crash risk as shown in Equations (7)–(9):


$$\text{Mediato}r_{i,t}=\delta_{0}+\delta_{1}AI_{i,t}+\sum \gamma_{k}\text{Contro}l_{i,t}+\mu_{i}+\gamma_{t}+\varepsilon_{i,t}$$ (7)

$$\begin{array}{c} \text{NCSKE}W_{i,t+1}=\delta_{0}+\delta_{1}AI_{i,t}+\delta_{2}\text{Mediato}r_{i,t}\\ +\sum \gamma_{k}\text{Contro}l_{i,t}+\mu_{i}+\gamma_{t}+\varepsilon_{i,t} \end{array}$$ (8)

$$\begin{array}{c} \text{DUVO}L_{i,t+1}=\delta_{0}+\delta_{1}AI_{i,t}+\delta_{2}\text{Mediato}r_{i,t}\\ +\sum \gamma_{k}\text{Contro}l_{i,t}+\mu_{i}+\gamma_{t}+\varepsilon_{i,t} \end{array}$$ (9)

In these models, $\text{Mediato}r_{i,t}$ represents the ICQ and DME for the *i*-th firm in the *t*-th year; the definitions of the other variables are the same as in models (1) and (2).

#### 4.4.1 The ICQ mechanism test

To examine the mechanism by which the AI mitigates the crash risk by enhancing enterprise ICQ, we select the DIB Internal Control Index as a proxy variable. This index comprehensively covers dimensions such as the enterprise’s internal environment, risk assessment, and oversight mechanisms, and serves as an authoritative indicator for measuring the efficiency of internal governance. To reduce differences in scale, the index (0–1,000) was divided by 100 before being included in the regression analysis.

The results in Table 7 show that: in column (1), the regression coefficient for the relationship between AI and the DIB Index is significantly positive at 1% level, confirming the role of AI technology in enhancing the quality of enterprise internal oversight; in Column (2), after incorporating the Dibo Index into the baseline model, the absolute value of the coefficient for AI adoption on the crash risk decreased, yet remained significant at the 1% level; furthermore, the regression coefficient for the Dibo Index was significantly negative, indicating that the quality of internal oversight played a significant mediating role. To ensure the robustness of the findings, this study employed the Bootstrap method (with 1,000 repeated samples) for testing; the mediation coefficients were separately 0.015^***^ (*Z* = −3.928, *p* = 0.00001) and 0.007^***^ (*Z* = −2.876, *p* = 0.004), passing the significance test. The above evidence indicates that enterprise AI can indeed effectively reduce crash risk by enhancing the ICQ, thereby confirming Hypothesis H2a.

**Table 7:** Mechanism test.

Variable	(1)	(2)	(3)	(4)	(5)	(6)
	ICQ_DIB index	NCSKEW	DUVOL	DME_NIE	NCSKEW	DUVOL
AI	0.021^***^	−0.021^**^	−0.015^***^	−0.093^***^	−0.015^***^	−0.012^*^
	(0.007)	(0.009)	(0.005)	(0.023)	(0.005)	(0.007)
ICQ_DIB index		−0.016^***^	−0.009^***^			
		(0.004)	(0.003)			
DME_NIE					0.015^**^	0.037^***^
					(0.007)	(0.009)
Ret	−0.691	−10.382^***^	−9.019^***^	0.081	−11.175^***^	−9.741^***^
	(0.761)	(0.827)	(0.525)	(1.492)	(1.116)	(0.701)
Sigma	−0.353	−5.605^***^	−2.593^***^	0.549	−6.062^***^	−2.763^***^
	(0.233)	(0.272)	(0.172)	(0.579)	(0.358)	(0.225)
Age	0.028	−0.242^***^	−0.170^***^	−0.671^***^	−0.242^***^	−0.162^***^
	(0.046)	(0.028)	(0.018)	(0.066)	(0.038)	(0.024)
Size	0.561^***^	0.042^***^	−0.006	0.132^***^	0.040^*^	−0.004
	(0.014)	(0.015)	(0.010)	(0.040)	(0.021)	(0.013)
Roe	0.477^***^	−0.046	−0.025	−0.999^***^	−0.068	−0.032
	(0.029)	(0.034)	(0.022)	(0.087)	(0.042)	(0.026)
Lev	0.334^***^	−0.032	0.019	−6.387^***^	−0.090	−0.014
	(0.049)	(0.056)	(0.036)	(0.146)	(0.075)	(0.047)
BM	−0.310^***^	−0.182^***^	−0.009	0.227^**^	−0.065	0.075^**^
	(0.039)	(0.044)	(0.028)	(0.110)	(0.060)	(0.038)
Cash	0.182^***^	0.175^***^	0.137^***^	5.698^***^	0.226^***^	0.165^***^
	(0.055)	(0.057)	(0.036)	(0.150)	(0.074)	(0.046)
Top1	0.002^**^	−0.001	−0.000	−0.003	−0.003^**^	−0.002^***^
	(0.001)	(0.001)	(0.001)	(0.002)	(0.001)	(0.001)
Inst	−0.000	0.003^***^	0.002^***^	−0.005^***^	0.004^***^	0.003^***^
	(0.001)	(0.001)	(0.000)	(0.002)	(0.001)	(0.001)
Big4	−0.029	−0.014	−0.019	−0.207^*^	−0.056	−0.051
	(0.037)	(0.046)	(0.029)	(0.122)	(0.063)	(0.039)
_cons	−4.170^***^	2.240^***^	−4.252^***^	−0.242	0.455^**^	2.829^***^
	(0.274)	(0.805)	(0.311)	(0.315)	(0.200)	(0.818)
Stkcd	Y	Y	Y	Y	Y	Y
Year	Y	Y	Y	Y	Y	Y
*N*	30,861	30,861	30,861	30,861	30,861	30,861
Adj. *R*^2^	0.916	0.108	0.117	0.622	0.111	0.123

#### 4.4.2 The DME mechanism test

To examine the mechanism by which artificial intelligence applications reduce the crash risk by enhancing corporate decision-making efficiency, we select the non-investment efficiency (NIE) value (the degree of deviation from investment efficiency) as a proxy indicator for decision-making efficiency. The extent of its deviation from the optimal boundary directly quantifies management’s inefficiency in information processing and resource allocation. Over-investment reflects the impulse to expand blindly in the absence of internal controls, while under-investment exposes decision-making limitations arising from financing constraints or excessive conservatism, leading to missed opportunities. The higher this indicator, the further the enterprise’s investment deviates from the optimal level and the lower its decision-making efficiency; conversely, a lower value indicates superior decision-making quality and serves as a classic benchmark for assessing the efficiency of resource allocation.

The results in Table 7 indicate that, in Column (3), the regression coefficient for AI on the non-investment efficiency value is significantly negative at the 1% level, confirming that AI technology significantly reduces investment errors through data-driven decision-making (Zhang et al., 2026). In Column (4), after incorporating this indicator into the baseline regression model, the absolute value of the regression coefficient for the enterprise’s AI application on the risk of a share price crash decreased compared with the baseline model, but remained significant at the 1% level; simultaneously, the regression coefficient for the non-investment efficiency value was significantly positive (i.e., the greater the deviation from optimal investment efficiency, the higher the risk of a crash). This result is consistent with recent research findings on AI-enhancing decision-making robustness (Ferrante, 2024). To further validate the robustness of the mediating pathway, this study employed the Bootstrap method (with 1,000 samples) for testing; the mediating effect coefficients were separately 0.016^***^ (*Z* = 4.54859, *p* = 0.000) and 0.009^***^ (*Z* = 3.963, *p* = 0.004), indicating robust significance. The above evidence strongly supports the hypothesis H2b, namely that AI does indeed effectively reduce the crash risk by optimizing the decision-making process and reducing investment deviations.

### 4.5 Heterogeneity test

#### 4.5.1 CEO digital expertise

A CEO with digital expertise can better capture strategic trends, unlocking AI’s governance potential to mitigate crash risk. Following Lu (2025), first, we define “CEO digital expertise” as an individual who has systematically majored in core digital disciplines—such as computer science, software engineering, electronic information, data science, and artificial intelligence—during their higher education (including associate’s, bachelor’s, master’s, and doctoral degrees). Second, during the data collection and cleaning process, we used executive resumes from the CSMAR database as a foundation. We manually cross-checked each entry against the annual reports of listed enterprises and performed rigorous “noise removal” to exclude non-academic short-term training programs completed by executives later in their careers, as well as general management master’s degrees that do not specify a major (such as general EMBA or MBA programs). This ensured that only samples with a truly solid academic background in science and engineering were retained. Finally, regarding coding rules, this study adopted a strict dummy variable assignment method: if a CEO’s major at any academic level included the aforementioned digital keywords, the digital professional competence variable was assigned a value of 1; if their entire academic background consisted solely of traditional disciplines (such as traditional management, history, or mechanical engineering), the variable was assigned a value of 0. Table 8, Columns (1)–(4) report these heterogeneity results. The mitigating influence of AI on crash risk is significantly more pronounced in enterprises with a digitally expert CEO. The results of the test for differences in coefficients between groups indicate that there are statistically significant differences in the regression coefficients. This suggests that executive digital expertise strengthens the inhibitory effect of AI applications on crash risk.

**Table 8:** Heterogeneity test.

Variable	CEO with digital expertise	CEO without digital expertise	CEO with digital expertise	CEO without digital expertise	Eastern	Central-Western	Eastern	Central-Western
	(1)	(2)	(3)	(4)	(5)	(6)	(7)	(8)
	NCSKEW	NCSKEW	DUVOL	DUVOL	NCSKEW	NCSKEW	DUVOL	DUVOL
AI	−0.018^*^	−0.017	−0.014^**^	−0.012	−0.022^**^	−0.016	−0.018^***^	−0.011
	(0.010)	(0.016)	(0.006)	(0.011)	(0.010)	(0.017)	(0.007)	(0.011)
Ret	−10.928^***^	−10.249^***^	−9.153^***^	−10.019^***^	−11.599^***^	−6.269^***^	−10.144^***^	−6.494^***^
	(0.960)	(1.602)	(0.614)	(1.011)	(0.990)	(1.546)	(0.631)	(0.973)
Sigma	−4.530^***^	−8.274^***^	−1.915^***^	−4.122^***^	−4.891^***^	−7.308^***^	−2.155^***^	−3.531^***^
	(0.313)	(0.537)	(0.200)	(0.340)	(0.326)	(0.518)	(0.208)	(0.326)
Age	−0.137^***^	−0.111^*^	−0.084^***^	−0.093^**^	−0.191^***^	−0.274^***^	−0.138^***^	−0.167^***^
	(0.031)	(0.062)	(0.020)	(0.039)	(0.032)	(0.056)	(0.020)	(0.036)
Size	0.083^***^	−0.062^**^	0.019	−0.077^***^	0.055^***^	0.002	0.003	−0.033^*^
	(0.019)	(0.028)	(0.012)	(0.018)	(0.020)	(0.032)	(0.013)	(0.020)
Roe	−0.093^**^	−0.077	−0.054^**^	−0.051	−0.047	−0.069	−0.018	−0.048
	(0.042)	(0.055)	(0.027)	(0.035)	(0.044)	(0.056)	(0.028)	(0.035)
Lev	−0.120^*^	0.334^***^	−0.053	0.285^***^	−0.143^**^	0.138	−0.036	0.068
	(0.067)	(0.104)	(0.043)	(0.066)	(0.072)	(0.109)	(0.046)	(0.069)
BM	−0.149^***^	−0.274^***^	0.014	−0.071	−0.152^***^	−0.168^**^	−0.004	0.005
	(0.054)	(0.077)	(0.035)	(0.048)	(0.055)	(0.085)	(0.035)	(0.054)
Cash	0.153^**^	0.173	0.142^***^	0.088	0.182^**^	0.109	0.130^***^	0.133^*^
	(0.067)	(0.113)	(0.043)	(0.071)	(0.071)	(0.111)	(0.045)	(0.070)
Top1	−0.001	−0.003^**^	−0.001	−0.001	0.000	−0.002	0.000	−0.001
	(0.001)	(0.002)	(0.001)	(0.001)	(0.001)	(0.002)	(0.001)	(0.001)
Inst	0.003^***^	0.005^***^	0.002^***^	0.003^***^	0.003^***^	0.004^***^	0.002^***^	0.002^***^
	(0.001)	(0.001)	(0.000)	(0.001)	(0.001)	(0.001)	(0.000)	(0.001)
Big4	−0.042	−0.015	−0.013	−0.043	−0.002	0.001	−0.017	0.015
	(0.068)	(0.066)	(0.043)	(0.041)	(0.062)	(0.079)	(0.040)	(0.050)
_cons	−1.466^***^	1.683^***^	−0.334	1.786^***^	−0.799^*^	0.662	0.105	1.058^**^
	(0.383)	(0.599)	(0.245)	(0.378)	(0.408)	(0.658)	(0.260)	(0.414)
Stkcd	Y	Y	Y	Y	Y	Y	Y	Y
Year	Y	Y	Y	Y	Y	Y	Y	Y
Differences in coefficients between groups	0.049^**^		0.025^**^		0.019^***^		0.042^**^	
*N*	19,959	10,902	19,959	10,902	20,567	10,294	20,567	10,294
Adj. *R*^2^	0.101	0.127	0.105	0.140	0.104	0.124	0.112	0.137

#### 4.5.2 Locational differences

Regional disparities in economic development and AI infrastructure across China may influence enterprise AI applications. Thus, we bifurcate the sample enterprise into Eastern and Central-Western regions. Table 8, Columns (5)–(8) show that AI’s inhibitory effect on crash risk is significantly more pronounced in the Eastern region. A test for differences in coefficients across groups counterpoised that there were statistically significant differences between the regression coefficients of the various groups, thereby validating the existence of a group effect. This likely stems from superior market mechanisms and stronger investor protection in Eastern China, which enhance AI’s governance efficacy and amplify its role in improving the information environment to mitigate risk.

## 5 Conclusions and recommendations

### 5.1 Conclusion

Using 2015–2024 Chinese A-share panel data, we empirically obtained that AI significantly reduces stock price crash risk. Mechanism tests show that AI mitigates risk by enhancing regulatory intensity and decision-making efficiency. In enterprises with a digitally expert CEO and those in Eastern China, the inhibitory effect is more pronounced. The results highlight AI’s vital role in improving enterprise governance and the information environment.

### 5.2 Recommendations

First, a precise policy incentive system should be established to integrate AI with the real economy. Governments should utilize fiscal and tax policies to lower transformation costs, embedding AI into supply chains to strengthen enterprises’ micro-foundations against crash risks. Second, enterprises must cultivate interdisciplinary management teams to enhance decision-making and disclosure efficiency while promoting regional resource sharing to mitigate information asymmetry. Third, regulators should refine information disclosure and valuation mechanisms for AI innovations. Establishing clear disclosure standards will help integrate AI depth into pricing systems, constraining managerial information-hiding and addressing crash risk at its source.

## Data Availability

The raw data supporting the conclusions of this article will be made available by the authors, without undue reservation.
